# Biocontrol Efficacy of a Seed-Derived *Streptomyces pseudogriseolus* YTU-S40 Against White Rot in Postharvest Grapes

**DOI:** 10.3390/microorganisms14081796

**Published:** 2026-08-14

**Authors:** Liyang Chu, Zeliang Guo, Cuiyao Zhang, Zheng Zhang, Lele Sun, Dongdong Meng, Yilin Luan, Juanjuan Liu

**Affiliations:** 1 Yantai Key Laboratory of Characteristic Agricultural Bioresource Conservation & Germplasm Innovative Utilization, College of Life Sciences, Yantai University, Yantai 264005, China; 2705403967@s.ytu.edu.cn (Z.G.); cuiyaozhang@163.com (C.Z.); 15065321439@163.com (Z.Z.); 15564286092@163.com (L.S.); mengdd@ytu.edu.cn (D.M.); liujj@ytu.edu.cn (J.L.); 2 School of Food and Biological Engineering, Yantai Institute of Technology, Yantai 264003, China

**Keywords:** *Streptomyces*, biocontrol efficacy, genome analysis, bioactive compounds, untargeted metabolomics

## Abstract

Grape white rot, a fungal disease caused by *Coniella diplodiella*, leads to the decay and abscission of leaves and fruits. This disease is recognized as one of the major fungal diseases affecting the grape industry, significantly impacting the quality and yield of grapes. In this study, a seed-derived *Streptomyces* strain with high biocontrol activity, designated YTU-S40, was isolated from the seeds of *Cnidium monnieri* (L.). Molecular phylogenetic identification revealed that this isolate belongs to *Streptomyces pseudogriseolus.* In vitro plate confrontation experiments demonstrated that YTU-S40 exhibited a robust inhibition rate of 76.2% against *C. diplodiella*. Furthermore, this strain exhibited relatively broad-spectrum antifungal activity, and genomic analysis predicted that its genome harbors 21 biosynthetic gene clusters responsible for secondary metabolite production. In vivo postharvest grape biocontrol assays verified that YTU-S40 significantly reduced the incidence of grape white rot from 94.4% to 1.9%. Untargeted metabolomic profiling suggested that YTU-S40 may produce bioactive antifungal compounds such as antimycins. Collectively, these findings suggest that YTU-S40 holds considerable promise for development as a biocontrol agent.

## 1. Introduction

Grape white rot, a fungal disease caused by *Coniella diplodiella*, is prevalent in temperate and tropical grape-growing regions worldwide [1]. Commonly referred to as “hail disease,” it thrives in high-temperature and high-humidity conditions, infecting all green tissues of grapevines, particularly the cluster rachis. Grape berries remain highly susceptible to infection from veraison to ripening. The pathogen’s conidia primarily disseminate through rain splash, entering grape tissues through wounds or microcracks. This disease significantly threatens the grape industry, typically leading to a 10–20% yield reduction in affected years [2]. In addition to lowering the market value of table grapes, mycotoxin residues in infected berries also impair the quality of wine and downstream grape-based processed products [3]. Current field management practices rely heavily on chemical fungicides; however, their excessive long-term application not only increases production costs but also drives the emergence of fungicide-resistant pathogens, raising concerns over pesticide residues, ecological damage, and food safety risks [4].

Biological control, a disease management method that utilizes naturally occurring organisms or their derivatives, has attracted extensive attention due to its environmental sustainability and ecological safety [5]. Unlike chemical pesticides, microbial biocontrol agents offer a promising eco-friendly alternative with a minimal environmental footprint. These antagonistic microorganisms—encompassing bacteria [6], fungi [7], and actinomycetes [8]—employ diverse strategies to suppress phytopathogens, ranging from direct antagonism, such as the secretion of hydrolytic enzymes and antibiotics, to indirect mechanisms such as niche competition and the induction of systemic resistance in host plants [9,10,11,12,13].

Antagonistic actinomycetes, particularly *Streptomyces*, are crucial components of the biocontrol system, playing a vital role in eco-friendly fungal disease management by producing diverse secondary metabolites. *Streptomyces* species are prevalent in various habitats, including soil, oceans, and plant tissues [14], and can produce a wide range of bioactive compounds with antifungal, antibacterial, and insecticidal activities [15]. In addition to direct antagonistic and parasitic activities, *Streptomyces* are able to induce systemic resistance and promote growth in plants [16].

Numerous studies have validated the biocontrol efficacy of *Streptomyces* against various fungal diseases. For instance, mupirocin produced by the root endophyte *Streptomyces lydicus* 6G-OA-10 disrupts the hyphal morphology of *Colletotrichum gloeosporioides* and activates host defense-related enzymes, achieving 97.36% postharvest control efficacy against anthracnose [17]. Similarly, the marine-derived *Streptomyces* sp. H4 produces dibutyl phthalate that effectively reduces postharvest strawberry decay while preserving fruit quality [18].

In the field of grape fungal disease management, *Streptomyces* strains exhibit notable biocontrol efficacy. For instance, *Streptomyces* sp. FX13 inhibits *Botrytis cinerea* through the synthesis of oligomycin A, providing protective effects against gray mold [19]. *Streptomyces noursei* C27 significantly reduces gray mold infection in grape berries [20], and combined biocontrol approaches using *Streptomyces* strains have achieved substantial reductions in downy mildew symptoms, comparable to those achieved by chemical fungicides [21]. However, despite these advancements, a critical research gap remains: the biocontrol potential of *Streptomyces* against grape white rot (*Coniella diplodiella*), an important fungal disease affecting postharvest grape quality, remains largely unexplored. Given the strict regulations on postharvest chemical fungicide residues, there is a pressing need to identify effective and eco-friendly biocontrol agents specifically tailored for white rot management.

In this study, the endophytic strain YTU-S40 was isolated from *Cnidium monnieri* (L.) and taxonomically identified as *Streptomyces pseudogriseolus* based on 16S rRNA gene sequencing. Strain YTU-S40 exhibits significant in vitro antagonistic activity against various phytopathogens. Notably, it demonstrates effective control in vivo against grape white rot caused by *Coniella diplodiella*. Therefore, this study aims to (i) isolate and identify strain YTU-S40, and determine its antagonistic effects on *C. diplodiella*; (ii) evaluate the biocontrol efficacy of strain YTU-S40 against postharvest grape white rot; and (iii) analyze the potential bioactive metabolites produced by the strain based on multi-omics analysis. By elucidating the biocontrol performance and the potential bioactive substances of strain YTU-S40, this study provides a highly promising microbial resource for the sustainable management of grape white rot.

## 2. Materials and Methods

### 2.1. Isolation and Purification of YTU-S40

The target strain was isolated from 50 healthy *Cnidium monnieri* (L.) seeds based on a modified protocol described by Chimwamurombe et al. [22]. First, the seeds were surface-sterilized with 75% ethanol, followed by treatment with sodium hypochlorite (NaClO), and then rinsed three times with sterile distilled water and dried. Subsequently, the dried samples were thoroughly ground with an appropriate volume of sterile water, and 200 μL of the resulting suspension was evenly spread onto isolation medium plates. After incubation at 28 °C for 7 days, colonies with distinct morphological characteristics (such as shape, color, and size) were observed and selected. The isolated single colonies were purified for 2–3 successive generations using the streak plate method and then preserved as glycerol stocks (20%, *v*/*v*) at −80 °C. The final rinse solution was used as a control and plated onto the isolation medium to verify the effectiveness of surface sterilization. Ultimately, 26, 0, 11, and 9 *Streptomyces* strains were isolated from the roots, stems, leaves, and seeds of *Cnidium monnieri* (L.), respectively.

### 2.2. Screening of Antagonistic Strains Against Coniella diplodiella

*C. diplodiella* was used as the indicator strain to evaluate the antifungal activity of the endophytic strains from *C. monnieri* (L.) using a plate confrontation assay [23]. The test strains were initially inoculated onto potato dextrose agar (PDA) plates as 8 mm-diameter mycelial plugs and incubated for three days. Subsequently, an 8 mm mycelial plug of the pathogen was inoculated at the center of each plate. Plates without antagonistic strains served as the blank controls. All plates were incubated at 28 °C for 96 h. Each treatment comprised three replicates, and the experiment was independently conducted three times. The data presented here are the means ± standard deviation (SD) of these three independent replicates. The inhibition rate of the test strain was calculated according to the method described by Rong et al. [24] using the following formula:

(1) Inhibition rate (%) = [(Colony radius of the control − Colony radius of the treatment)/(Colony radius of the control − Initial plug radius)] × 100%

### 2.3. Identification of YTU-S40

#### 2.3.1. Morphological and Physiological Characterization of YTU-S40

The morphological characteristics of YTU-S40 were determined using a Gram staining kit (Solarbio, Beijing, China). Culture media were prepared according to the method described by Wang et al. [25]. The colony morphology, aerial mycelium, and substrate mycelium pigmentation of YTU-S40 were observed after incubation on ISP3 to ISP7 media at 28 °C for 14 days. Additionally, the growth of YTU-S40 was evaluated under various temperatures, pH levels, NaCl concentrations, and in the presence of different sole carbon or nitrogen sources [26].

#### 2.3.2. 16S rRNA Sequencing and Phylogenetic Tree Construction

The genomic DNA of the antagonistic strain was extracted using the TIANamp Genomic DNA Kit (DP302, Tiangen, Beijing, China). Using the extracted genomic DNA as a template, the 16S rRNA gene was amplified via PCR with the primers 27F (5′-AGAGTTTGATCCTGGCTCAG-3′) and 1492R (5′-TACGACTTAACCCCAATCGC-3′). The amplified target fragments were detected via agarose gel electrophoresis, subsequently purified, and sent to Sangon Biotech (Shanghai, China) for sequencing; the obtained sequences were submitted to the NCBI database. Closely related type strain sequences were retrieved from the NCBI database and aligned using MEGA 11. A phylogenetic tree was constructed using the neighbor-joining method, and bootstrap values (expressed as percentages) were calculated based on 1000 replicates.

### 2.4. Scanning Electron Microscopy (SEM) Analysis

Cell samples of YTU-S40 were cultured in liquid medium at 28 °C and 180 rpm for 5 days for SEM observation of the morphological characteristics of the strain. In addition, *C. diplodiella* samples from the control group and the confrontation group containing YTU-S40 (see Section 2.2) were collected to evaluate the impact of the antagonistic strain on the microstructural growth of the fungus. Subsequently, samples were fixed in 2.5% glutaraldehyde in 0.1 M phosphate buffer (pH 7.2) for 4 h at 4 °C, washed, post-fixed in 1% osmium tetroxide for 2 h, dehydrated through a graded ethanol series (30%, 50%, 70%, 90%, 100%; 10 min for each step), critical-point dried, and sputter-coated with gold to a thickness of ~10 nm [27]. Finally, the prepared samples were observed and photographed using a Hitachi Regulus 8100 scanning electron microscope (SEM; Hitachi, Tokyo, Japan).

### 2.5. Detection of Extracellular Enzyme Production and Growth-Promoting Ability

To evaluate the capacity of YTU-S40 to produce extracellular enzymes and promote plant growth, specific media for detecting amylase, cellulase, xylanase, glucanase, siderophores, and indole-3-acetic acid (IAA) were prepared according to previously reported methods [28]. YTU-S40 was inoculated onto the respective media and incubated for 5–7 days. Following incubation, indicators such as the formation of transparent zones (hydrolysis halos) were observed. A standard curve was established using a microplate reader (BioTek Instruments, Inc., Winooski, VT, USA) to quantitatively measure the IAA production of the strain under exogenous tryptophan conditions. All experiments were performed in three independent replicates.

### 2.6. Genome Sequencing, Annotation, and Comparative Analysis

#### 2.6.1. Genome Sequencing

Whole-genome sequencing and assembly of YTU-S40 were performed by Novogene Co., Ltd. (Beijing, China) [29]. Paired-end sequencing (PE150) was conducted on the Illumina NovaSeq platform. After quality filtering of the raw data, the clean reads were separately assembled using SOAPdenovo 2.04, SPAdes 1.3.7, and ABySS 1.3.7. The resulting assemblies were integrated and optimized using CISA, and the remaining gaps were closed using GapCloser 1.12. Contigs shorter than 500 bp or with a sequencing depth below 0.35-fold of the average depth were filtered out to obtain the final genome assembly.

#### 2.6.2. Genome Annotation and Secondary Metabolite Analysis

Based on the assembled genome, protein-coding genes were predicted using GeneMarkS 4.17. Functional annotation was performed via BLASTp 2.12.0 alignment (E-value ≤ 1 × 10^−5^) against the Cluster of Orthologous Groups (COG), Gene Ontology (GO), and Kyoto Encyclopedia of Genes and Genomes (KEGG) databases [30]. In addition, bioinformatics analysis of secondary metabolite biosynthetic gene clusters (BGCs) within the genome was conducted using antiSMASH 5.1. The carbohydrate-active enzymes encoded in the genome were identified via alignment against the CAZy database (https://www.cazy.org/, accessed on 28 January 2025).

#### 2.6.3. Genome-Based Taxonomic Identification

Genome sequences of the reference type strains related to YTU-S40 (identified in the phylogenetic analysis in Section 2.3.2) were downloaded from the NCBI database. The average nucleotide identity (ANI) and digital DNA–DNA hybridization (dDDH) values between YTU-S40 and the reference strains were calculated using the online tool OrthoANI (https://www.ezbiocloud.net/tools, accessed on 15 December 2025) and the Genome-to-Genome Distance Calculator (GGDC v2.1) with Formula 2 [17], respectively.

### 2.7. Determination of the Broad-Spectrum Antifungal Activity of YTU-S40

Following the plate confrontation assay described in Section 2.2, the inhibitory activity of YTU-S40 against ten additional plant-pathogenic fungi—*Trichothecium roseum*, *Colletotrichum graminicola*, *Botryosphaeria* spp., *Rhizoctonia cerealis*, *Colletotrichum viniferum*, *Botrytis cinerea*, *Phytophthora nicotianae*, *Fusarium proliferatum*, *Fusarium graminearum*, and *Helminthosporium maydis*—was evaluated. The endpoint for calculating the inhibition rate of each fungus was determined as the time point when the mycelium of the corresponding control plate nearly covered the entire plate surface.

### 2.8. Evaluation of In Vitro Antifungal Activity of Cell-Free Culture Filtrate and Extracts

YTU-S40 was first cultured in seed culture medium at 180 rpm for 2 days, and then inoculated into fermentation medium at a 6% (*v*/*v*) inoculation rate and cultured at 180 rpm for 7 days. The fermentation broth was collected and centrifuged at 8000 rpm for 10 min. The supernatant was filtered through a 0.22 μm membrane to completely remove the mycelia, yielding the cell-free culture filtrate, which was subsequently mixed into PDA plates at final concentrations of 4%, 8%, 12%, and 16% (*v*/*v*); mycelial plugs of *C. diplodiella* were inoculated at the center of these plates. PDA plates without the cell-free culture filtrate served as the blank control. After all plates were incubated for 4 days, the diameter of the fungal mycelium was measured, and the inhibition rate of the cell-free culture filtrate was calculated as described in Section 2.2. In addition, an equal volume of ethyl acetate (1:1, *v*/*v*) was added to the cell-free culture filtrate and extracted twice. The organic phase was concentrated using a rotary evaporator to obtain the ethyl acetate extract, which was then mixed into PDA plates at final concentrations of 0.25%, 0.5%, 1%, and 1.5% (*v*/*v*). The subsequent measurement and calculation methods for the inhibition rate of the extract were consistent with those used for the cell-free culture filtrate. All experiments were performed in three independent replicates.

### 2.9. Control Efficacy of YTU-S40 Fermentation Extract on Detached Grape Fruits

All plant materials (Kyoho grapes) used in this study were purchased from a local supermarket in Yantai, Shandong Province. Grape fruits with uniform maturity and size were strictly selected. All fruits were surface-disinfected, washed, and air-dried before being used to evaluate the in vivo biocontrol efficacy of YTU-S40 [31]. A sterile needle was used to create wounds of uniform size (3 mm in diameter, 1 mm in depth) on the surface of each grape. Subsequently, 20 μL of sterile water or the fermentation extract was added to treat the wounds in the control (CK) and treatment groups, respectively, followed by air-drying at room temperature. Then, 20 μL of *C. diplodiella* spore suspension (1 × 10^7^ spores/mL) was inoculated onto the wounds of all grape fruits, and they were placed in a plant growth chamber. The disease incidence and lesion size were evaluated at 0.5, 1, 2, and 3 days post-inoculation. Each treatment consisted of three replicates containing eighteen grapes, and the experiment was conducted independently three times.

### 2.10. Untargeted Metabolomic Profiling of the YTU-S40 Fermentation Extract

To profile the metabolites within the ethyl acetate extract prepared as described in Section 2.8, an untargeted metabolomics approach was employed at Novogene Co., Ltd. (Beijing, China). The extraction of metabolites followed established protocols [32]. Chromatographic separation was achieved on a Vanquish UHPLC system (Thermo Fisher Scientific, Bremen, Germany) equipped with a Hypersil Gold column (100 × 2.1 mm, 1.9 μm). A 12 min linear gradient at a flow rate of 0.2 mL/min was applied, utilizing eluent A (0.1% formic acid in water) and eluent B (methanol). The gradient program was configured as follows: 0–1.5 min at 2% B; 1.5–3 min at 2–85% B; 3–10 min at 85–100% B; 10–10.1 min at 100–2% B; and maintained at 2% B until 12 min. Mass spectrometric detection was executed using an Orbitrap Q Exactive™ HF mass spectrometer (Thermo Fisher Scientific) operating in both positive and negative ion modes. Key MS parameters included a spray voltage of 3.5 kV, a capillary temperature of 320 °C, a sheath gas pressure of 35 psi, an auxiliary gas flow of 10 L/min, an S-lens RF level of 60, and an auxiliary gas heater temperature of 350 °C.

Following data acquisition, raw MS data were processed using the XCMS software (version 3.12.0) package for peak alignment and extraction. Putative metabolite identification was accomplished by matching MS/MS spectra and adduction information against a high-quality MS/MS spectral database. Finally, functional annotation and pathway enrichment of the identified metabolites were conducted by mapping them to specialized databases, including KEGG.

### 2.11. Statistical Analysis

All data are presented as the mean ± standard deviation. Statistical differences between groups were evaluated using a *t*-test in GraphPad Prism 10.1.2. For experiments involving multiple groups, multiple *t*-tests were conducted. The thresholds for statistical significance were set at * *p* < 0.05, ** *p* < 0.01, and *** *p* < 0.001.

## 3. Results

### 3.1. In Vitro Antifungal Activity of YTU-S40 Against C. diplodiella

**Figure 1:**
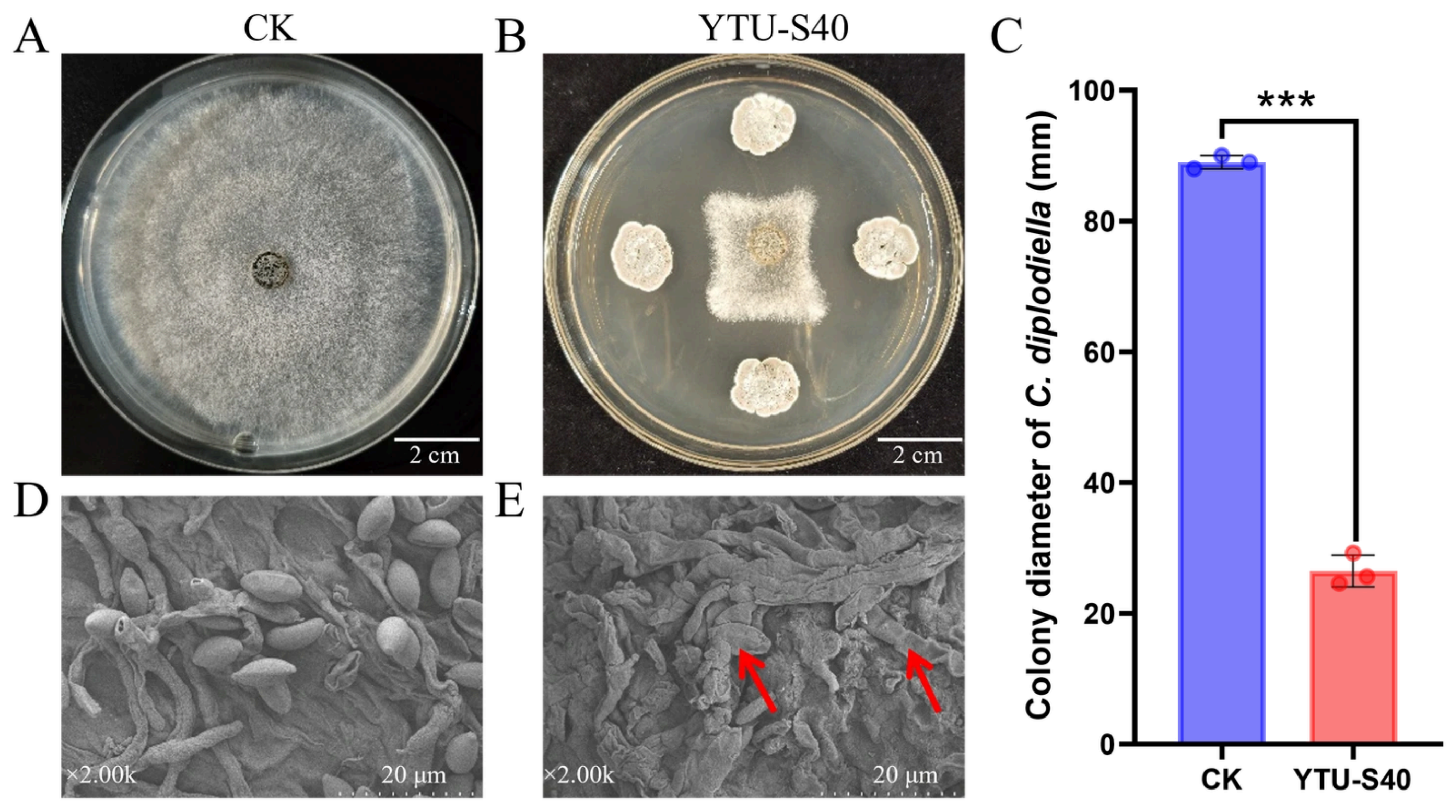
In vitro antifungal activity of YTU-S40 against *C. diplodiella*: (**A**) Control group. (**B**) Inhibitory effect of YTU-S40 on the mycelial growth of *C. diplodiella* on PDA plates. (**C**) The colony diameter of *C. diplodiella* was determined after incubation for 4 days. (**D**,**E**) SEM observations of *C. diplodiella* mycelia (scale bars = 20 μm) from the control group and YTU-S40 treatment group, respectively. Red arrows indicate hyphal breakage, fragmentation, or spore deformation. Data are presented as the means ± standard deviations (SD, error bars) of three independent replicates. Differences between CK and YTU-S40 were significant at *p* < 0.001 (***).

Plate confrontation assays revealed that YTU-S40 significantly inhibited the mycelial growth of *C. diplodiella* (Figure 1A,B). Following a 4-day co-cultivation, the colony diameter of *C. diplodiella* in the control group reached 89.0 mm, whereas it measured only 26.5 mm in the YTU-S40 treatment group. This result demonstrates the prominent inhibitory effect of YTU-S40 on *C. diplodiella*’s growth on PDA medium, resulting in a maximum inhibition rate of 76.2% (Figure 1C). Scanning electron microscopy (SEM) observations showed that *C. diplodiella* in the control group maintained typical and healthy mycelial morphology, characterized by a slender mycelium with oval, plump, and well-defined spores (Figure 1D). In contrast, *C. diplodiella* mycelia in the YTU-S40 treatment group exhibited rough and irregular surfaces, with partial mycelial fracture and fragmentation; additionally, the spores lost their original shapes and became deformed, exhibiting surface wrinkles and depressions (Figure 1D). These findings underscore the potent antifungal activity of YTU-S40. Although SEM provided qualitative evidence of hyphal damage, future studies could quantify morphological changes through image analysis to strengthen the conclusions.

### 3.2. Morphological Characterization

**Figure 2:**
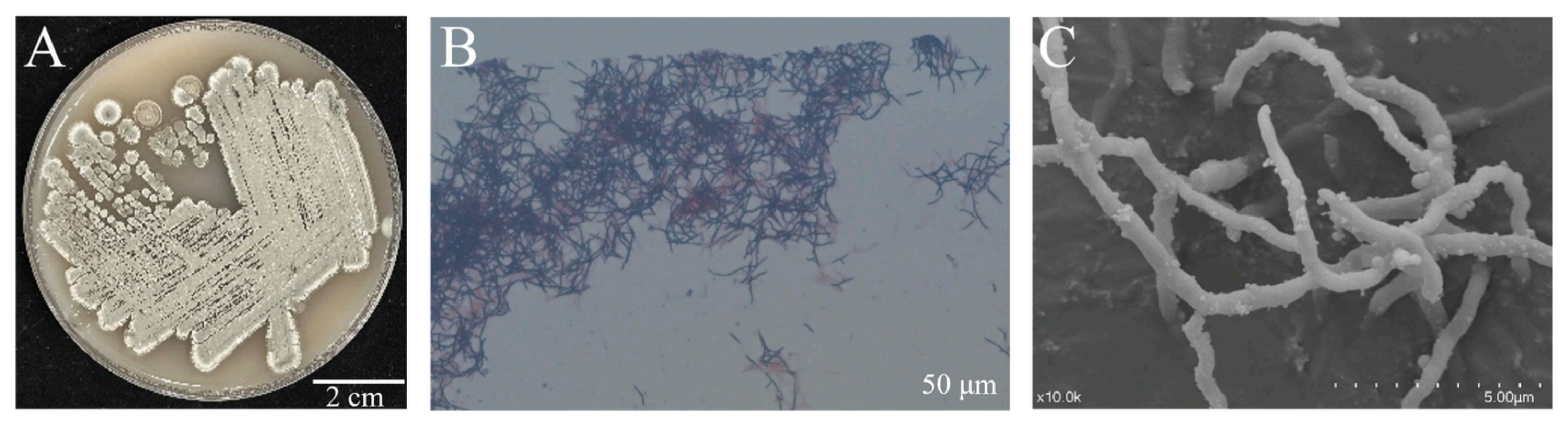
Morphological characteristics of YTU-S40: (**A**) Colony morphology on ISP3, with a 2 cm scale bar. (**B**) Gram staining, with a 50 μm scale bar. (**C**) Scanning electron microscopy images of YTU-S40, with a 5 μm scale bar.

YTU-S40 exhibited typical morphological characteristics consistent with the genus *Streptomyces* when cultured on five media, among which ISP3 medium was selected for subsequent phenotypic characterization. On ISP3 medium, the strain grew vigorously and formed gray-brown aerial mycelium; its colonies had regular margins, a slightly convex center, and dense mycelium (Figure 2A). Gram staining showed that YTU-S40 stained purple, confirming that this strain is a Gram-positive bacterium. Scanning electron microscopy observations revealed that the strain produced straight and curved filamentous hyphae that formed a dense interwoven network, which is consistent with the typical morphological characteristics of *Streptomyces* species (Figure 2B,C). In contrast, the aerial hyphae on the remaining four media appeared white, light brown, yellow, and light gray. Notably, colonies cultured on ISP4 and ISP5 were smaller, with sparser mycelia, indicating suboptimal growth of the strain on these two media (Figure S1).

### 3.3. Physiological and Biochemical Characteristics

Physiological and biochemical experiments demonstrated that YTU-S40 could utilize xylose, fructose, and other carbohydrates as sole carbon sources but could not metabolize inositol or sorbitol (Table S1). Concurrently, YTU-S40 could use alanine, proline, and arginine as sole nitrogen sources for growth. This strain grew at temperatures of 20–40 °C and pH 7–11, and it tolerated NaCl concentrations up to 7%, indicating its tolerance to multiple environmental stresses.

### 3.4. Detection of Extracellular Enzyme Synthesis and Growth-Promoting Ability

**Figure 3:**
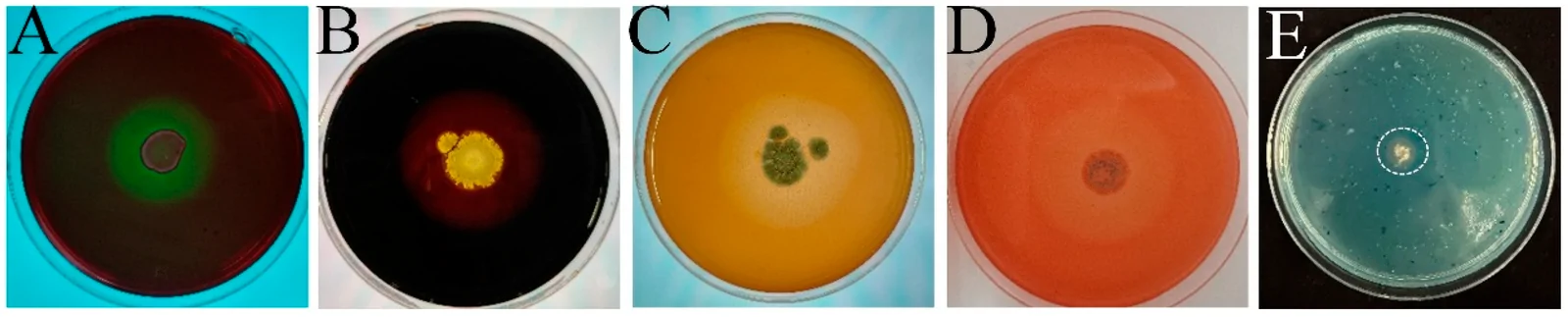
Detection of extracellular enzymes and siderophore production in YTU-S40. Cellulase (**A**), amylase (**B**), xylanase (**C**), glucanase (**D**), and siderophore biosynthesis (**E**) were determined.

Enzymatic screening on multiple diagnostic media confirmed that YTU-S40 could secrete extracellular hydrolases, such as cellulase, amylase, xylanase, and glucanase (Figure 3A–D). Notably, xylanase and glucanase exhibited exceptionally high hydrolytic activity, with clear zone ratios of 2.7 and 3.1, respectively. This highlights the significant potential of YTU-S40 for the biocontrol of plant diseases. In addition, YTU-S40 could produce siderophores and indole-3-acetic acid (IAA), with the concentration of IAA measured at 22.8 mg/L (Figure 3E and Figure S2), suggesting its capacity to promote plant growth.

### 3.5. Taxonomic Identification of YTU-S40

**Figure 4:**
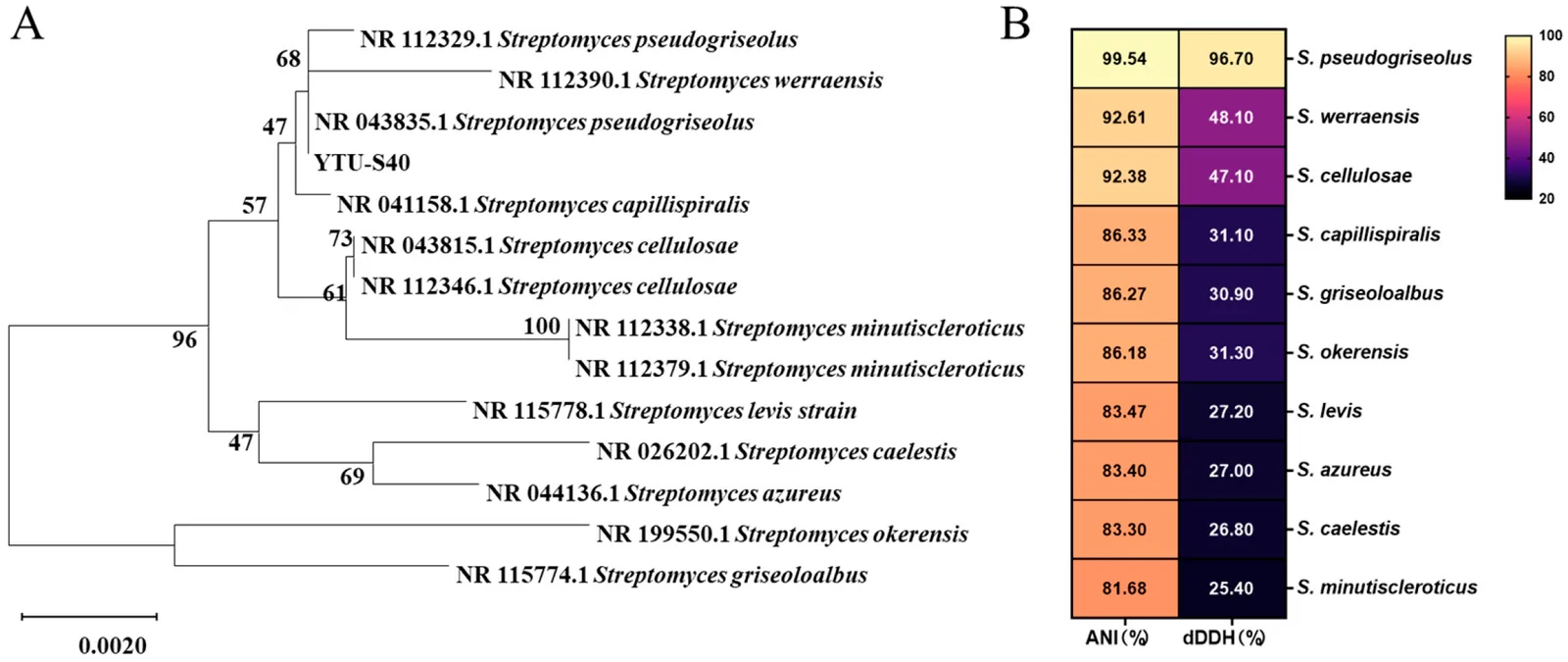
Taxonomic identification of YTU-S40: (**A**) Phylogenetic tree of YTU-S40 based on its 16S rRNA sequence. (**B**) OrthoANI and dDDH values between YTU-S40 and closely related strains.

The 16S rRNA sequence of YTU-S40 (1514 bp, GenBank accession number: PZ571461) was aligned with sequences of validated type strains deposited in the NCBI database. Thirteen closely related *Streptomyces* strains were chosen for phylogenetic analysis. A phylogenetic tree was constructed using the MEGA 111 software, with bootstrap values calculated with 1000 replicates (Figure 4A). Genome taxonomic analysis yielded an average nucleotide identity (ANI) value of 99.54% and a digital DNA–DNA hybridization (dDDH) value of 96.7% between YTU-S40 (GenBank accession number JCAGWX000000000) and *S. pseudogriseolus* (GenBank accession number: NR 043835.1) (Figure 4B and Table S2); both values surpassed the species delineation thresholds. Consequently, by integrating phenotypic, 16S rRNA, and genomic evidence, YTU-S40 was identified as *S. pseudogriseolus*.

### 3.6. Broad-Spectrum Antifungal Activity of YTU-S40

**Figure 5:**
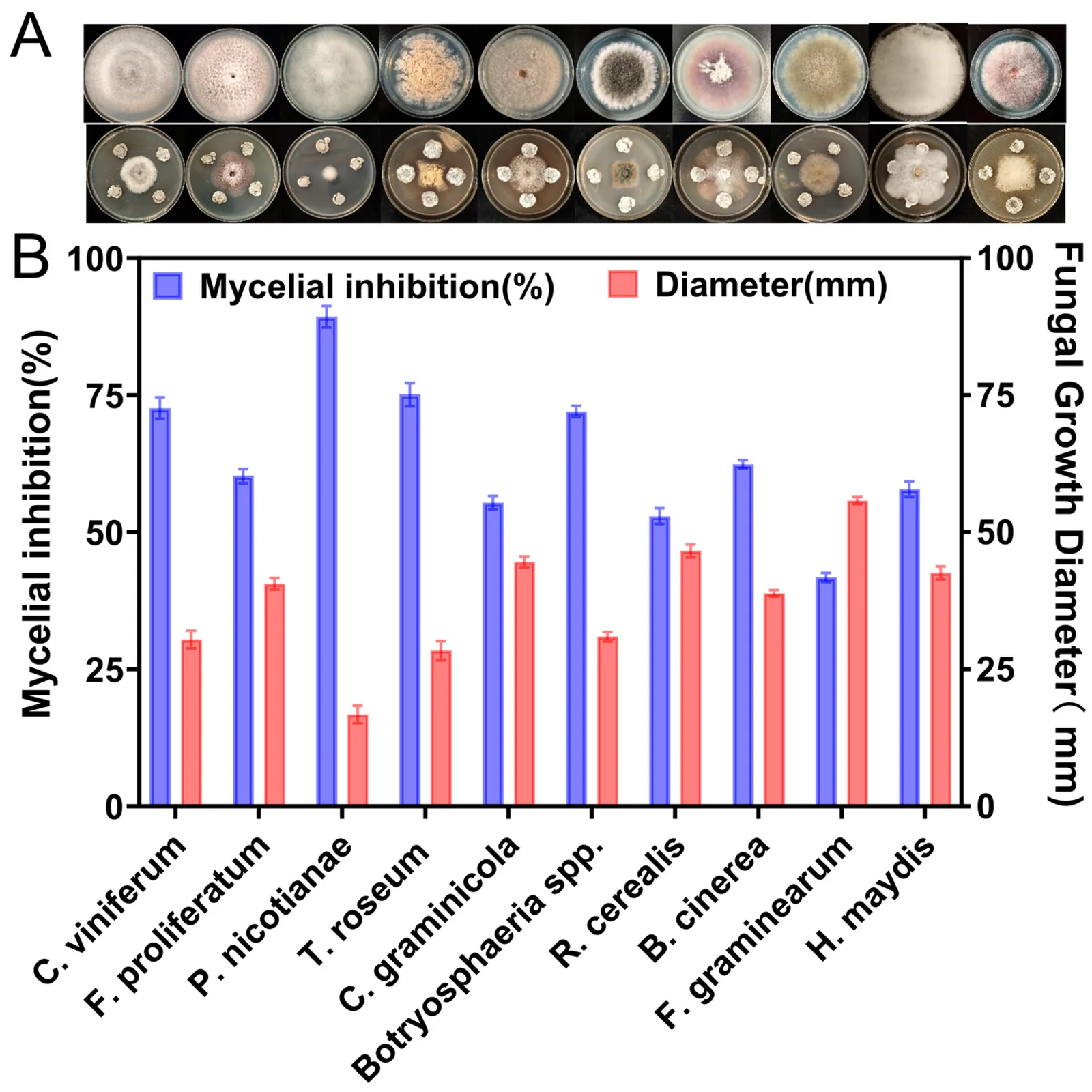
Broad-spectrum antifungal activity of YTU-S40: (**A**) Antifungal activity of YTU-S40 on PDA plates. (**B**) Mycelial inhibition rates (%) of YTU-S40 against seven plant-pathogenic fungi; error bars represent standard deviation (SD).

Plate confrontation experiments were used to assess the antagonistic activity of YTU-S40 against ten phytopathogenic fungi and confirm its broad-spectrum antifungal capability. The findings revealed that YTU-S40 exerted varying inhibitory effects on the tested fungi, with inhibition rates ranging from 41.7% to 89.3% (Figure 5). Notably, the highest inhibition rate of 89.3% was observed against *P. nicotianae*, followed by a 75.1% inhibition rate against *T. roseum*. These results suggest that YTU-S40 exhibits a broad antifungal spectrum, indicating its potential for development as a broad-spectrum antifungal agent.

### 3.7. Whole-Genome Analysis of YTU-S40

**Figure 6:**
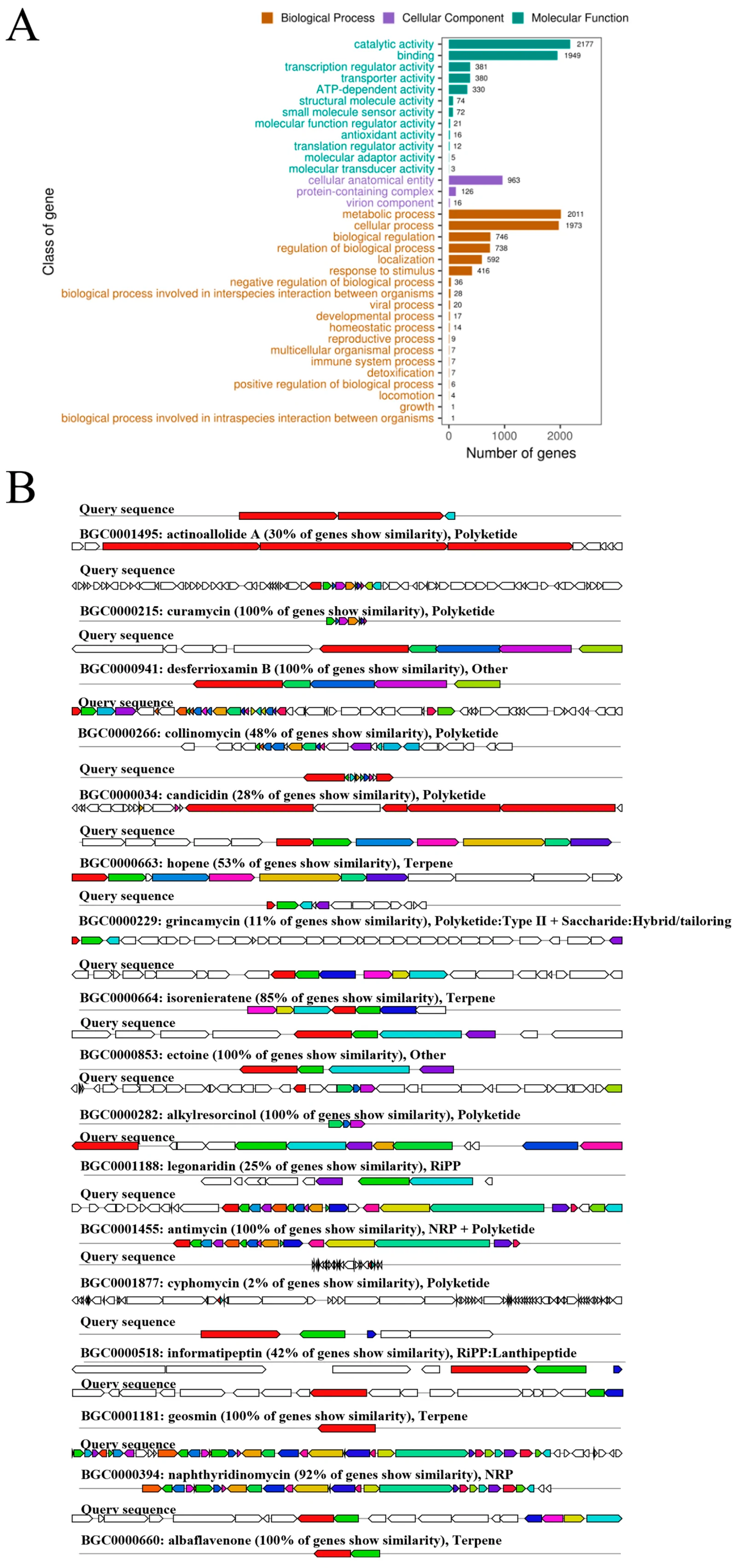
Whole-genome sequencing and annotation of YTU-S40: (**A**) GO annotation; (**B**) secondary metabolite gene clusters.

Given the notable antifungal activity of YTU-S40, whole-genome sequencing and annotation were conducted. The assembled genome spans 7,330,134 bp, with a GC content of 72.67%, and harbors 6491 coding genes, accounting for 85.93% of the overall genome length (Figure S3); additionally, 68 tRNAs, 8 rRNAs, and 22 sRNAs were predicted. Gene Ontology (GO) functional annotation classified 3950 genes, of which 2177 were associated with catalytic activity and binding (Figure 6A). Furthermore, 2511 and 4677 genes were identified in the KEGG and COG databases, respectively (Figures S4 and S5). Carbohydrate-Active enZYmes (CAZy) database screening identified 453 carbohydrate-active enzymes in YTU-S40, predominantly comprising 176 glycoside hydrolases (GHs) and 23 carbohydrate esterases (CEs) (Figure S6). This genomic observation aligns with the previously obtained results regarding various extracellular enzyme activities.

A total of 21 secondary metabolite biosynthetic gene clusters were predicted on the YTU-S40 genome using the AntiSMASH 5.1. Seven of them exhibited 100% similarity with well-characterized gene clusters responsible for the biosynthesis of secondary metabolites such as geosmin, antimycin, and ectoine [33]. In addition, four gene clusters corresponded to uncharacterized metabolites with unknown structures and biological functions (Figure 6B and Table S3). Furthermore, four gene clusters exhibited less than 30% similarity to functionally validated clusters, suggesting that YTU-S40 may produce novel secondary metabolites. These findings demonstrate that this strain is a promising microbial resource for the discovery of unreported bioactive compounds.

### 3.8. Controlling Effect of YTU-S40 Extracts on Hyphal Growth of C. diplodiella

**Figure 7:**
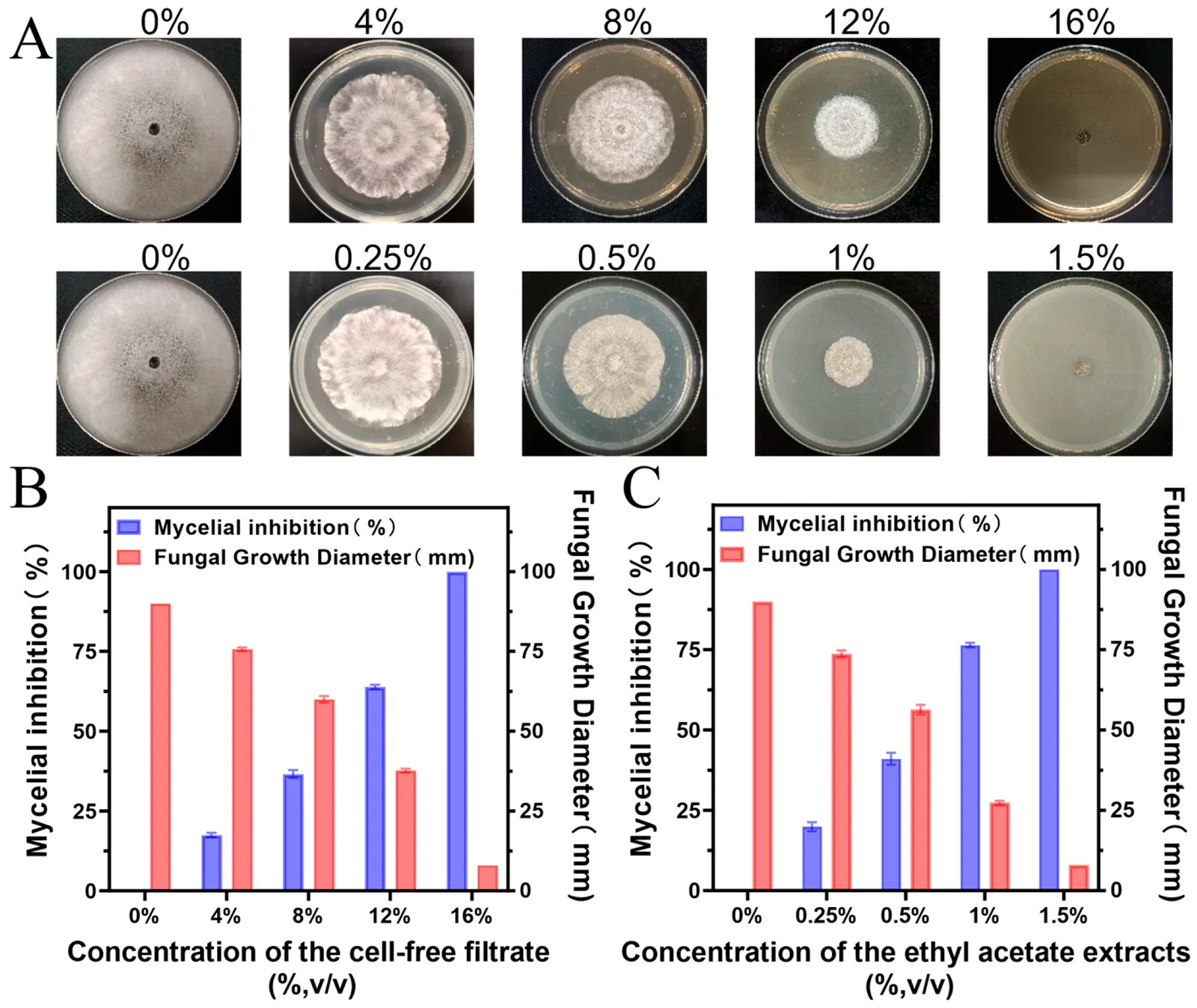
Controlling effect of YTU-S40 extracts on hyphal growth of *C. diplodiella*: (**A**) Effects of cell-free culture filtrate at different concentrations (4%, 8%, 12%, and 16%) and ethyl acetate extracts at different concentrations (0.25%, 0.5%, 1%, and 1.5%) on the mycelial growth of *C. diplodiella*. (**B**,**C**) Inhibitory activity of cell-free filtrate and ethyl acetate extracts on *C. diplodiella*, respectively.

To further investigate the inhibitory effects of active substances from YTU-S40 on the growth of the plant-pathogenic fungus *C. diplodiella*, cell-free culture filtrate and ethyl acetate extracts were supplemented into PDA medium at different concentration ratios. The findings revealed a dose-dependent inhibition of *C. diplodiella* growth with increasing proportions of cell-free culture filtrate. Notably, the growth of *C. diplodiella* was nearly completely inhibited when the filtrate addition ratio reached 16% (*v*/*v*) in PDA medium (Figure 7A,B). Similarly, increasing the supplementation concentration of ethyl acetate extracts gradually enhanced the antifungal effect. Specifically, with the addition of 0.25%, 0.5%, 1%, and 1.5% ethyl acetate extracts, the inhibition rates against *C. diplodiella* reached 19.9%, 41.1%, 76.4%, and 100%, respectively (Figure 7C). These results suggest that YTU-S40 produces antifungal secondary metabolites during fermentation, which are efficiently extracted using ethyl acetate. Highly concentrated metabolite solutions can effectively suppress the growth of the plant-pathogenic fungus *C. diplodiella*.

### 3.9. Protection of Postharvest Grapes from White Rot by YTU-S40

**Figure 8:**
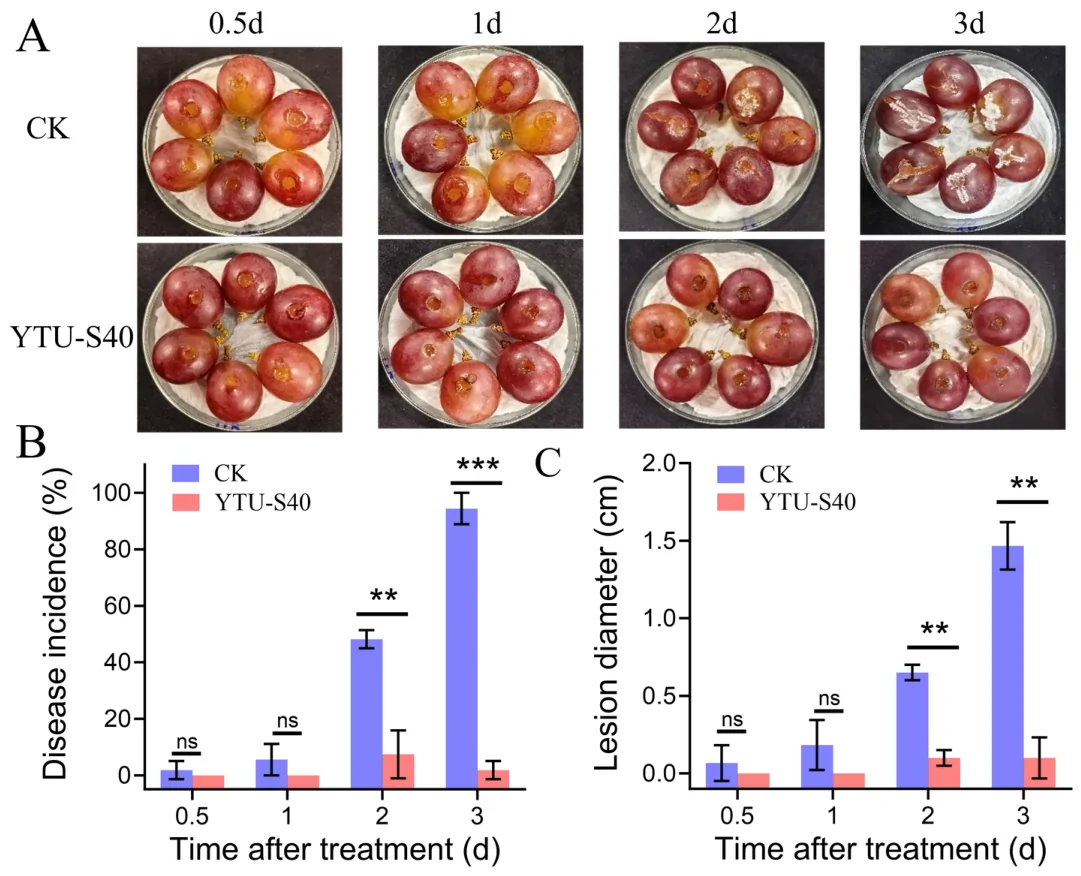
The biological control effect of YTU-S40 on grape white rot: (**A**) The inhibitory effect of YTU-S40 on grape white rot at different time points, (**B**) Comparison of disease incidence rates among different treatment groups. (**C**) Comparison of lesion sizes on grape berries among different treatment groups. ns indicates no significant difference. Asterisks (**), and (***) indicate statistically significant differences at *p* < 0.01 and *p* < 0.001, respectively. Values shown are means of triplicate determinations.

In vivo experiments were conducted to evaluate the biocontrol efficacy of ethyl acetate extracts from YTU-S40 against grape white rot. The results indicated that the extract treatment significantly reduced disease incidence and lesion expansion compared with the control group. At three days post-inoculation, the incidence rate reached 94.4% in the control group, whereas the treatment group exhibited a markedly low incidence rate of only 1.9% (Figure 8A,B). Regarding lesion development, fruits treated with ethyl acetate extracts displayed a lesion diameter of 0.1 cm, representing only 6.8% of that observed in the control group (Figure 8C). These findings suggest that the ethyl acetate extracts of YTU-S40 effectively control grape white rot, highlighting the potential of this strain to yield novel compounds for the prevention of postharvest fungal diseases.

### 3.10. Analysis of Active Metabolites in Fermentation Extracts by Untargeted Metabolomics

**Figure 9:**
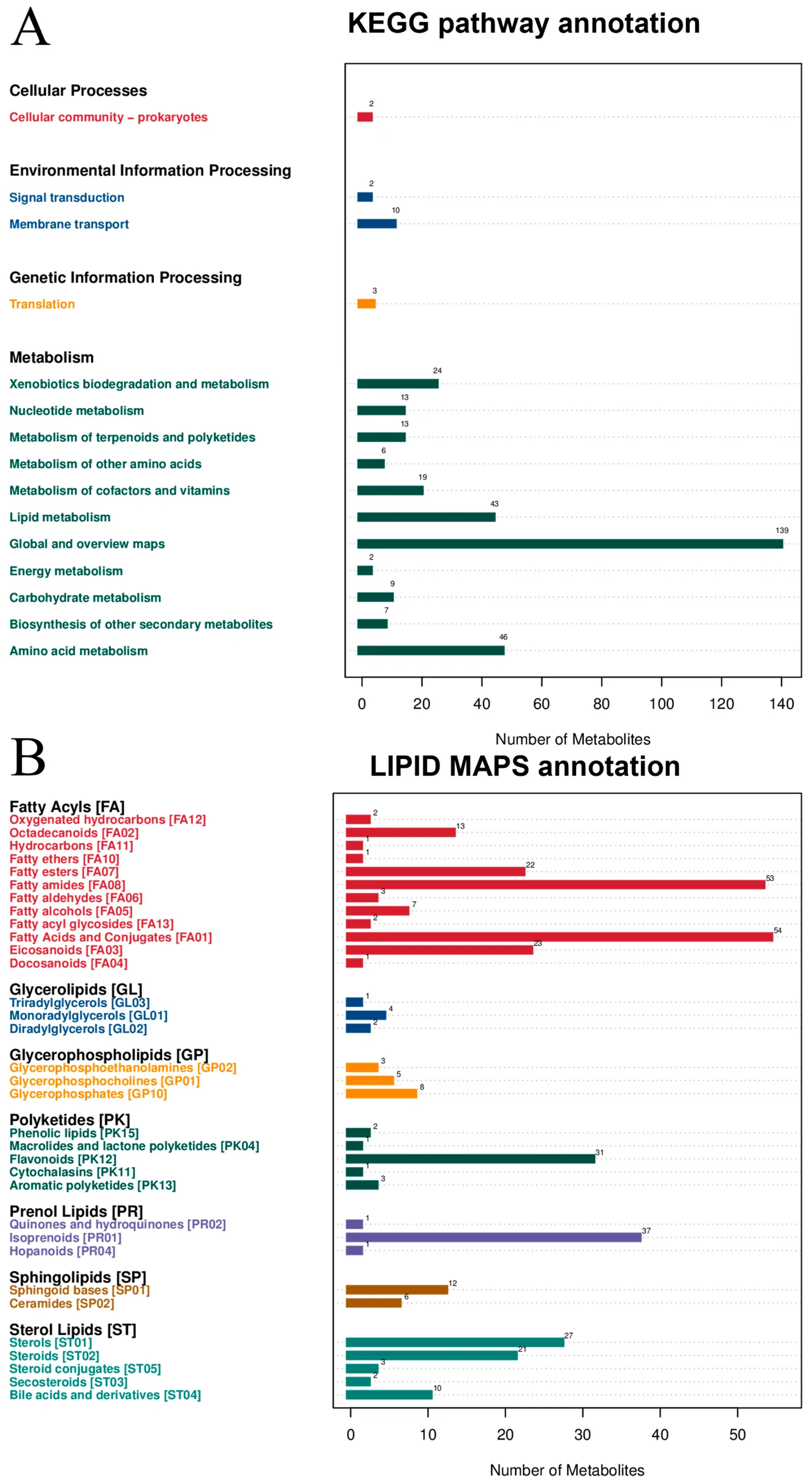
Analysis of active metabolites in fermentation extracts by untargeted metabolomics in positive ion mode: (**A**) KEGG pathway annotation. (**B**) LIPID MAPS annotation.

Untargeted metabolomics analysis identified 2606 metabolites in positive ion mode and 1281 metabolites in negative ion mode (Dataset S1). KEGG functional annotation indicated that most of the metabolites detected in positive ion mode were associated with global and overview maps (Figure 9A). Additionally, 13 metabolites were involved in the metabolism of terpenoids and polyketides, while seven metabolites participated in the biosynthesis of other secondary metabolites. For negative ion mode, two metabolites were annotated to the metabolism of terpenoids and polyketides, while six metabolites were linked to the biosynthesis of other secondary metabolites (Figure S7). Furthermore, several key bioactive compounds predicted via genomic analysis, including antimycin and ectoine, were successfully verified in this metabolomic profiling.

Further annotation against the LIPID MAPS database identified various compounds with putative antifungal activity, including macrolides, flavonoids, coumarins, and isoprenoids [33,34] (Figure 9B). Intriguingly, metabolomic profiling also detected several plant growth-regulating phytohormones, such as indole-3-butyric acid, gibberellin A3, and isopentenyladenosine, which are well-established plant growth-promoting regulators. Therefore, YTU-S40 is not only an antagonistic antifungal agent effective against plant fungal diseases but may also function as a biostimulant to regulate plant growth. These results elucidate the antifungal and growth-promoting mechanisms of YTU-S40, underscoring its potential as a valuable microbial agent in advancing green agricultural practices.

## 4. Discussion

Grape white rot, a severe postharvest disease, causes significant economic losses. Due to their favorable safety and environmental sustainability, biocontrol strategies have garnered increasing attention. *Streptomyces* represents a promising reservoir for biocontrol agents, as strains of this genus synthesize abundant bioactive secondary metabolites with remarkable antibacterial and antifungal activities [35]. For instance, wuyiencin, derived from *Streptomyces albus* var. *wuyiensis*, significantly reduces the severity of grape gray mold and delays resistance development in *Botrytis cinerea* [36]. In this study, the strain YTU-S40 was isolated and identified as a potential biocontrol agent (BCA) against grape white rot. By integrating phenotypic characterization and whole-genome comparative analysis, YTU-S40 was taxonomically identified as *S.pseudogriseolus*. Notably, this strain exhibited broad-spectrum antifungal activity against various plant-pathogenic fungi.

Genome sequencing revealed that YTU-S40 contains numerous conserved biosynthetic gene clusters (BGCs), including nonribosomal peptide synthetases (NRPSs), polyketide synthases (PKSs), and terpenes (Supplementary Table S3). Sequence alignment against the antiSMASH database predicted a total of 21 BGCs potentially encoding secondary metabolites. Among them, the BGCs responsible for compounds such as ectoine, albaflavenone, alkylresorcinol, and antimycin showed high similarity to previously characterized BGCs. Ectoine serves as an osmoprotectant that not only facilitates microbial adaptation to salinity fluctuations but also exerts antifungal effects by disrupting fungal cell membrane structures [37,38]. Albaflavenone, a tricyclic sesquiterpene antibiotic, exhibits significant inhibitory activity against multiple plant-pathogenic fungi [39]. Alkylresorcinols inhibit fungal growth by interfering with the lipid composition of fungal cell membranes [40]. As a respiratory chain inhibitor, antimycin exerts its antifungal effects by blocking the fungal electron transport chain, thereby inhibiting energy metabolism [41]. Consequently, YTU-S40 possesses the metabolic capability to synthesize a suite of bioactive metabolites that collectively endow this strain with powerful antifungal capacity. Notably, the current genome assembly relies solely on Illumina short-read sequencing. While sufficient for species delineation and preliminary BGC identification, this approach has inherent limitations in resolving highly repetitive regions. Therefore, complete reconstruction of complex BGCs may be fragmented, and long-read sequencing will be required in future studies to fully elucidate these biosynthetic pathways.

Untargeted metabolomics bridges “genome annotation” and “metabolite validation,” serving as a core technical approach for elucidating the antimicrobial mechanisms of microorganisms and discovering novel natural products [42,43,44]. In this study, ectoine, alkylresorcinol, and antimycin were detected via untargeted metabolomics, which matched genomic predictions and partially clarified the potential antifungal compounds of YTU-S40. Furthermore, additional antimicrobial components, such as coumarins and isoprenoids, were also detected in the metabolome. Previous studies have demonstrated that coumarins exert antifungal effects by compromising fungal cell membrane integrity, impairing mitochondrial function, and interfering with energy metabolism [34]. As a prominent coumarin derivative, osthole inhibits the MCR-1 enzyme, modulates host immunity, and disrupts fungal metabolic processes [45]. Collectively, these results indicate that YTU-S40 is capable of synthesizing a diverse arsenal of compounds with known antifungal properties to effectively combat fungal pathogens.

Furthermore, multiple phytohormones and plant growth-promoting metabolites were detected in the metabolomic profile, including cytokinins, auxins, gibberellins, brassinosteroids, and phytosiderophores. Trans-zeatin, an active cytokinin, regulates the proliferation and differentiation of procambial cells and cooperates with the auxin-mediated signaling network to determine organ morphogenesis and developmental processes in plants [46]. In terms of auxins, indole-3-butyric acid (IBA) serves as a storage form of the endogenous auxin indole-3-acetic acid (IAA). IBA is converted into IAA via β-oxidation in peroxisomes, providing localized auxin gradients to fine-tune root architecture and adaptive responses to environmental cues [47]. Additionally, indole-3-pyruvic acid is another biosynthetic precursor for IAA, which binds to TIR1/AFB receptors, promoting the degradation of Aux/IAA repressors and thereby releasing auxin response factors (ARFs) to regulate plant tropisms and organogenesis [48]. Gibberellins A3 and A4 (GA3 and GA4) are the primary endogenous active gibberellins in plants, maintaining GA homeostasis through the DELLA protein pathway and feedback regulatory mechanisms, thereby regulating critical developmental stages such as seed germination, stem elongation, and floral organ development [49,50]. Collectively, these results indicate that YTU-S40 combines robust antifungal properties with significant potential as a plant growth-promoting agent.

Over the past two years, research on the application of *Streptomyces* in the biocontrol of grape fungal diseases has expanded from in vitro inhibition assays to postharvest fruit and field-scale trials, significantly enhancing the credibility of its practical application. For instance, the rhizosphere strain *Streptomyces* LWT20 demonstrated effective control against *Botrytis cinerea*-induced gray mold on postharvest grapes, with its extract significantly inhibiting lesion expansion and maintaining fruit quality [51]. Compared to current *Streptomyces* biocontrol research primarily targeting gray mold and downy mildew, YTU-S40 exhibits unique advantages in several dimensions. First, its target disease is grape white rot (*C.a diplodiella*), a major grape disease that has received relatively little attention in current *Streptomyces* biocontrol research. White rot frequently causes 10–20% yield losses in years with high temperatures and rainfall [52]. YTU-S40 demonstrated a high inhibition rate of 76.2% against *C. diplodiella* during in vitro plate confrontation assays and reduced the incidence rate from 94.4% to 1.9% in detached fruit assays, showing excellent target specificity and potential application value. Second, regarding metabolic profiles, the genome of YTU-S40 was predicted to contain 21 secondary metabolite biosynthetic gene clusters, and metabolomics confirmed the production of antimycin, ectoine, coumarins, and various plant hormones (e.g., IBA and GA3). This “antifungal and growth-promoting” metabolic spectrum is distinct from some *Streptomyces* preparations that rely primarily on a single or few antibiotics, potentially offering advantages in delaying resistance development and providing growth promotion [21,51]. Finally, YTU-S40 was isolated as a seed endophyte from a medicinal plant; compared to rhizosphere- or soil-derived strains, its endophytic origin suggests unique potential for plant colonization and host interaction, providing new insights for developing “endophytic biocontrol agents” for grapevine diseases [52]. In conclusion, YTU-S40 is distinguished from current biocontrol research objects by its target disease spectrum, metabolic diversity, and ecological niche; it fills the gap in biocontrol resources for grape white rot and holds promise for development as a multifunctional biocontrol agent with both disease-suppressive and growth-promoting capabilities.

Although YTU-S40 has demonstrated excellent efficacy in detached fruit assays, several key limitations remain in transitioning from laboratory to field applications. First, existing work is primarily based on in vitro grape fruit models and has not systematically evaluated the stability of its efficacy in complex field environments. Field studies on grape diseases have shown that, even when a single strain extract exhibits high inhibition rates under indoor conditions, its efficacy is often significantly reduced in the field [21]. Therefore, YTU-S40’s actual field efficacy, colonization dynamics, and its impact on non-target microbial communities in vineyards need to be validated through multi-point and multi-season field trials. Second, commercial applications face common challenges regarding shelf-life and the stability of active ingredients. Many antifungal compounds are susceptible to environmental degradation under field conditions, including ultraviolet radiation, oxidation, and pH fluctuations [8]. Therefore, formulation strategies—such as microencapsulation, adsorption onto carriers, or emulsion systems—are often required in order to enhance the stability and shelf-life of *Streptomyces*-based biocontrol agents [8]. For YTU-S40, the predicted production of antimycins and other antifungal metabolites underscores the need to evaluate the stability of these compounds under different fermentation and formulation conditions. Third, integrating YTU-S40 into existing vineyard management systems is critical for large-scale application. Exploring the compatibility and synergistic effects of YTU-S40 fermentation extracts with existing fungicides (e.g., strobilurins, triazoles), while focusing application during critical susceptible stages (e.g., veraison to maturity) in combination with rain-shelter cultivation and proper pruning, will establish a comprehensive control system centered on YTU-S40 following a “biological priority, chemical assistance” approach. Finally, future research should focus on (1) optimizing fermentation processes for scalability and cost control, (2) determining the residual dynamics and safe intervals of major active ingredients on fruit surfaces, and (3) assessing compatibility with commonly used vineyard fungicides and fertilizers. Through this systematic work, YTU-S40 can be transitioned from a laboratory isolate to a practical vineyard biocontrol product.

## 5. Conclusions

The strain *S. pseudogriseolus* YTU-S40 was isolated from *C. monnieri* (L.), exhibiting high antagonistic activity against the causal pathogen of grape white rot. Through multi-omics analysis, this strain was predicted to synthesize diverse antifungal secondary metabolites (e.g., antimycin) as well as various plant hormones and growth-promoting compounds. This dual capability of inhibiting pathogens while promoting plant growth addresses the unstable control efficacy commonly observed for individual antagonistic bacteria in complex field environments. Consequently, this strain offers a practical and sustainable approach to reducing reliance on chemical pesticides in vineyards. Future research should focus on optimizing the liquid fermentation process for this strain, assessing its field efficacy under diverse ecological conditions, and evaluating its compatibility with commonly used fungicides. These future investigations will be to evaluate the potential for transitioning this strain from laboratory settings to field applications.

## Data Availability

The original contributions presented in this study are included in the article/Supplementary Material. Further inquiries can be directed to the corresponding authors.

## Supplementary Materials

The following supporting information can be downloaded at: https://www.mdpi.com/article/10.3390/microorganisms14081796/s1, Figure S1: Morphological characteristics of strain YTU-S40 on different culture media; Figure S2: Standard curve of indole-3-acetic acid (IAA); Figure S3: Distribution of gene lengths on the genome of strain YTU-S40; Figure S4: KEGG annotation of strain YTU-S40; Figure S5: COG annotation of strain YTU-S40; Figusre S6: Carbohydrate-Active enZYmes Database annotation of strain YTU-S40; Figure S7: Analysis of active metabolites in fermentation extracts by untargeted metabolomics in negative ion mode. (A) KEGG pathway annotation. (B) LIPID MAPS annotation; Table S1: Cultural, physiological, and biochemical characteristics of YTU-S40; Table S2: dDDH and ANI values between strain YTU-S40 and its closely related strains; Table S3: Secondary metabolite gene clusters of the YTU-S40 predicted by AntiSMASH; Dataset S1: Meta-intensity data of YTU-S40 fermentation extract.
