# Correction: Chu et al. Biocontrol Efficacy of a Seed-Derived *Streptomyces pseudogriseolus* YTU-S40 Against White Rot in Postharvest Grapes. *Microorganisms* 2026, *14*, 1796

**DOI:** 10.3390/microorganisms14102177

**Published:** 2026-09-28

**Authors:** Liyang Chu, Zeliang Guo, Cuiyao Zhang, Zheng Zhang, Lele Sun, Dongdong Meng, Yilin Luan, Juanjuan Liu

**Affiliations:** 1 Yantai Key Laboratory of Characteristic Agricultural Bioresource Conservation & Germplasm Innovative Utilization, College of Life Sciences, Yantai University, Yantai 264005, China; 2705403967@s.ytu.edu.cn (Z.G.); cuiyaozhang@163.com (C.Z.); 15065321439@163.com (Z.Z.); 15564286092@163.com (L.S.); mengdd@ytu.edu.cn (D.M.); liujj@ytu.edu.cn (J.L.); 2 School of Food and Biological Engineering, Yantai Institute of Technology, Yantai 264003, China

In the original publication [1], Reference 48 has been replaced with a more appropriate reference as follows:

Enders, T.A.; Strader, L.C. Auxin activity: Past, present, and future. *Am. J. Bot.* **2015**, *102*, 180–196. https://doi.org/10.3732/ajb.1400285.

The citation and numbering of all references remain unchanged. The authors state that the scientific conclusions are unaffected. This correction was approved by the Academic Editor. The original publication [1] has also been updated.
