# Supplementary material for: Biocontrol Efficacy of a Seed-Derived Streptomyces pseudogriseolus YTU-S40 Against White Rot in Postharvest Grapes
Source: Microorganisms. 2026 Aug 14;14(8):1796. doi: 10.3390/microorganisms14081796 (PMC13515182; doi:10.3390/microorganisms14081796)
Supplement: Supplementary file 1 [file microorganisms-14-01796-s001.zip › 20260814-S40-SM.pdf]

Supplementary Materials

Biocontrol Efficacy of a Seed-Derived *Streptomyces pseudogriseolus* YTU-S40 Against White Rot in Postharvest Grapes

Liyang Chu [1,*,†], Zeliang Guo [1,†], Cuiyao Zhang [1], Zheng Zhang [1], Lele Sun [1], Dongdong Meng [1], Yilin Luan [2,*] and Juanjuan Liu [1]

[1] Yantai Key Laboratory of Characteristic Agricultural Bioresource Conservation & Germplasm Innovative Utilization, College of Life Sciences, Yantai University, Yantai, 264005, China

[2] School of Food and Biological Engineering, Yantai Institute of Technology, Yantai, 264003, China

*Corresponding author: Liyang Chu, E-mail: chuliyang@ytu.edu.cn

Yilin Luan, E-mail: luanyilin@yitsd.edu.cn

† These authors contributed equally to this work.

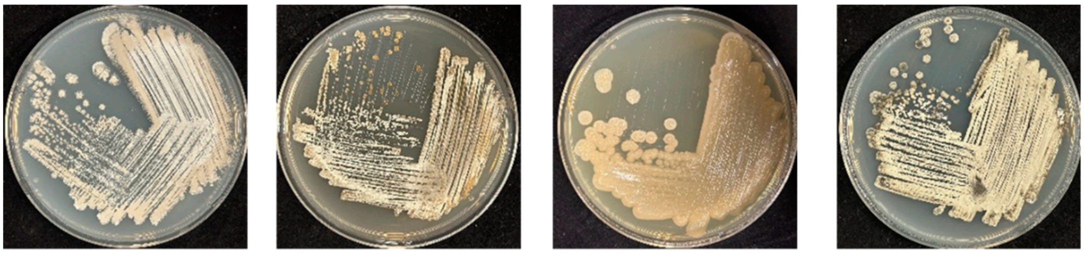

Figure S1. Morphological characteristics of strain YTU-S40 on different culture media.

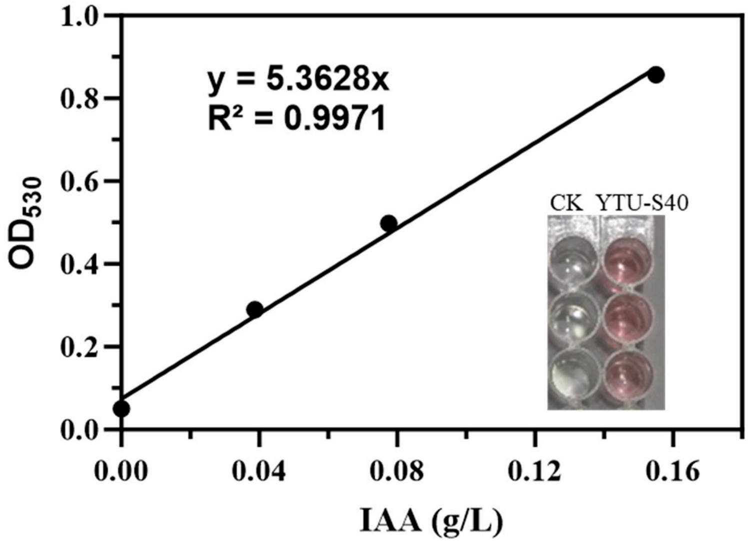

Figure S2. Standard curve of indole-3-acetic acid（IAA）.

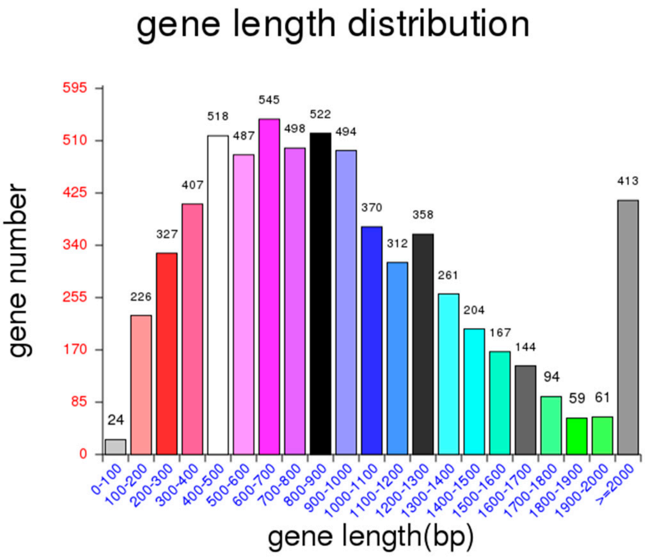

Figure S3. Distribution of gene lengths on the genome of strain YTU-S40.

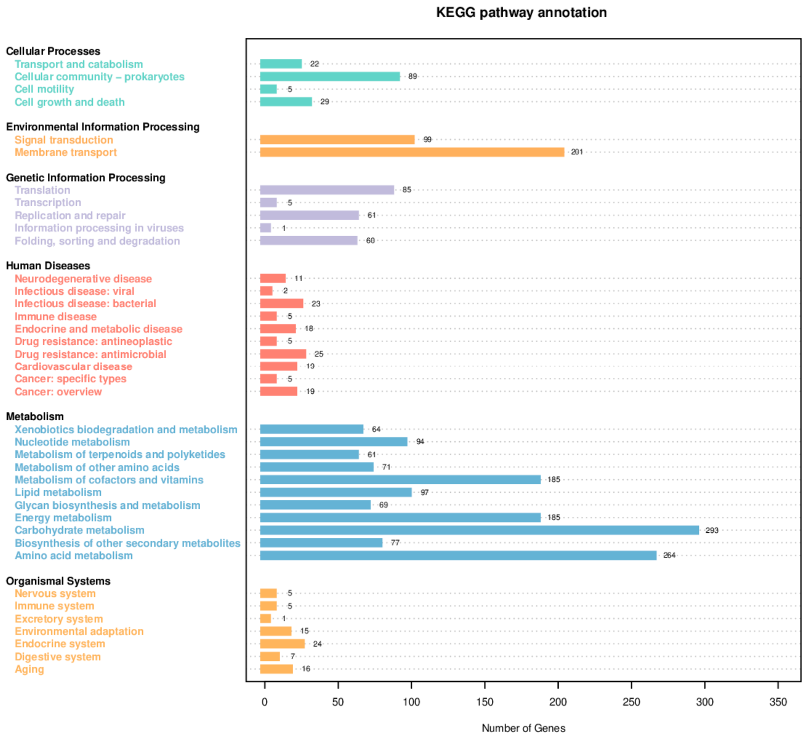

Figure S4. KEGG an-notation of strain YTU-S40.

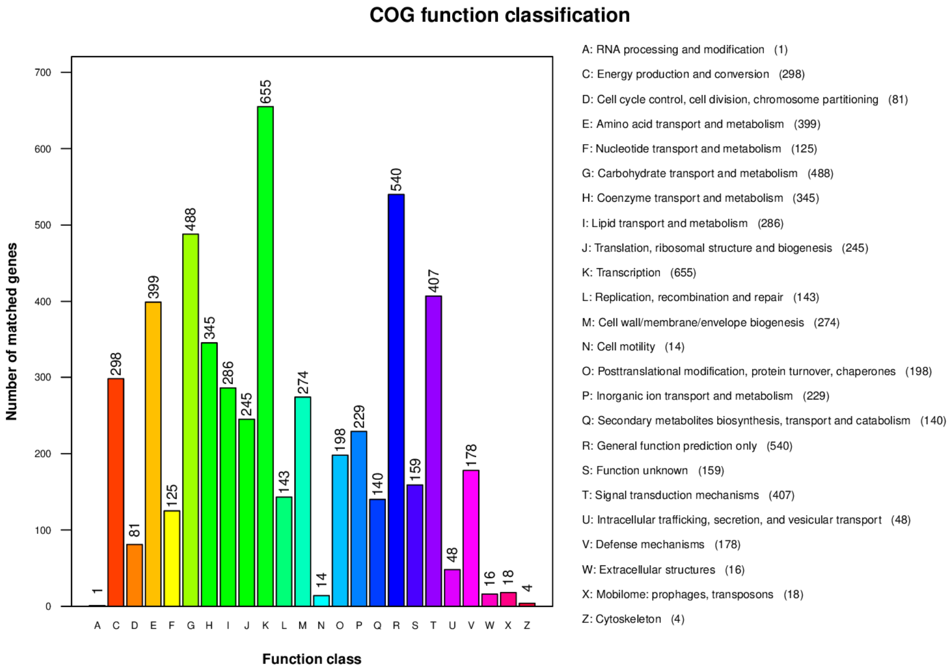

Figure S5. COG annotation of strain YTU-S40.

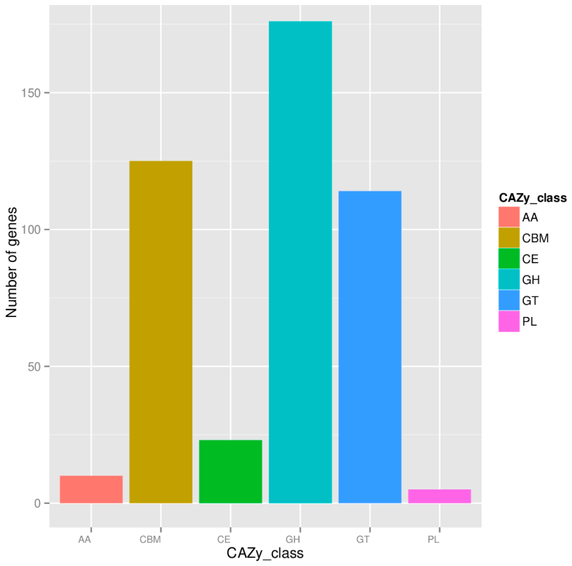

Figure S6. Carbohydrate-Active enZYmes Database annotation of strain YTU-S40.

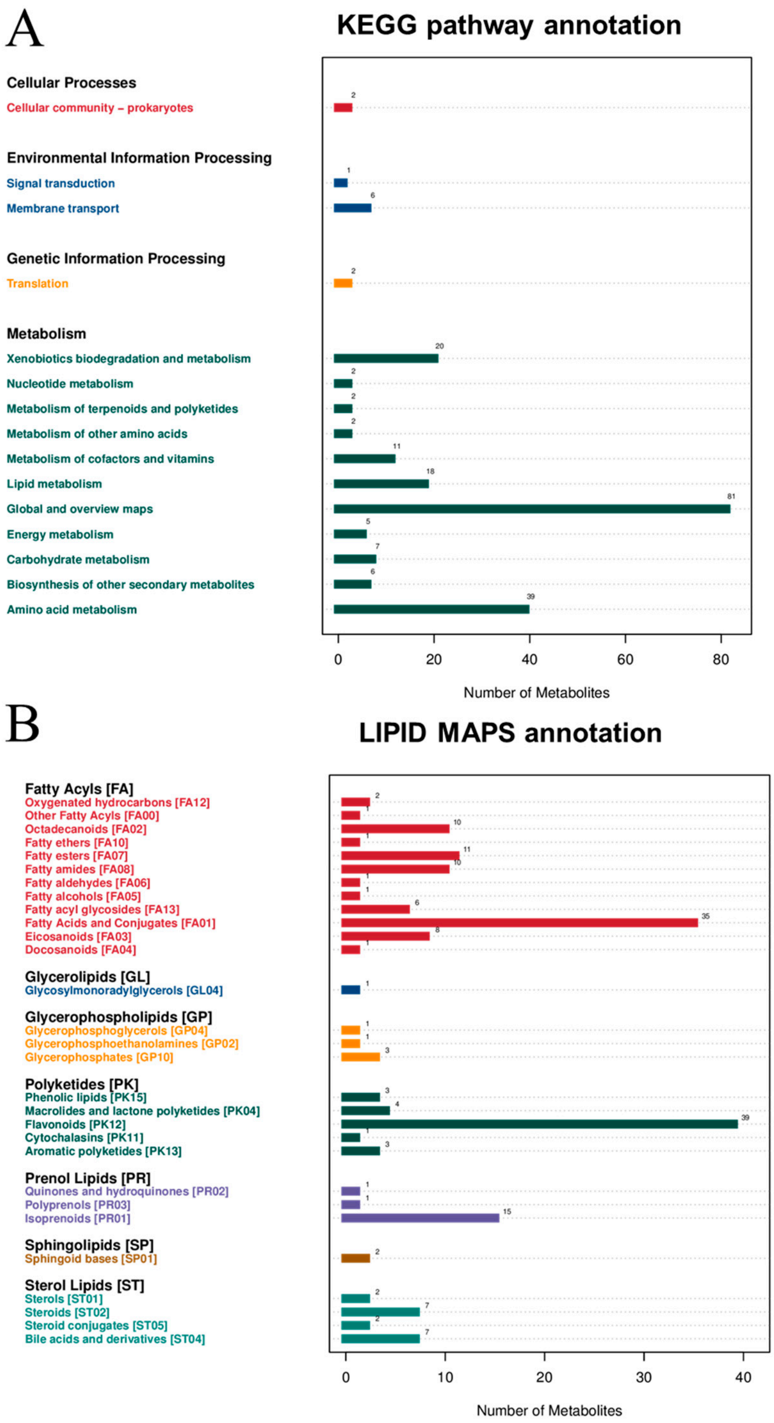

Figure S7. Analysis of active metabolites in fermentation extracts by untargeted metabolomics in negative ion mode. **(A)** KEGG pathway annotation. **(B)** LIPID MAPS annotation.

Table S1. Cultural, physiological, and biochemical characteristics of YTU-S40.

Identification items	Results
**Carbon Source Utilization**	
Inositol	-
Sorbitol	-
Mannitol	-
Maltose	+
Arabinose	+
Xylose	+
Fructose	+
Rhamnose	-
Sucrose	+
**Nitrogen Source Utilization**	
Glycine	+
Alanine	+
Proline	+
monosodium glutamate	+
Arginine	+
KNO_3	+
$(NH_4)_2SO_4$	+
**Growing conditions**	
Temperature	20-40℃
pH	7-11
NaCl	0-7%

Table S2 dDDH and ANI values between strain YTU-S40 and its closely related strains.

**Strain**	**dDDH（%）**	**ANI（%）**
Streptomyces azureus ATCC 14921	27	83.4
Streptomyces caelestis DSM 40084	26.8	83.3
Streptomyces capillispiralis DSM 41695	31.1	86.33
Streptomyces cellulosae NPDC057399	47.1	92.38
Streptomyces griseoloalbus JCM 4480	30.9	86.27
Streptomyces levis JCM 6924	27.2	83.47
Streptomyces minutiscleroticus JCM 4790	25.4	81.68
Streptomyces okerensis DSM 116494	31.3	86.18
Streptomyces pseudogriseolus JCM 4416	96.7	99.54
Streptomyces werraensis O57	48.1	92.61

Table S3. Secondary metabolite gene clusters of the YTU-S40 predicted by AntiSMASH.

Similar metabolite	Similarity (%)	Length (bp)
grincamycin	11	13226
geosmin	100	22157
informatipeptin	42	10216
isorenieratene	85	24105
alkylresorcinol	100	41182
cyphomycin	2	24547
antimycin	100	47825
desferrioxamin B	100	11773
collinomycin	48	53075
curamycin	100	72510
albaflavenone	100	20924
ectoine	100	10399
legonaridin	25	20579
naphthyridinomycin	92	70504
candicidin	28	38146
actinoallolide A	30	31429
hopene	53	14980
